# Pulmonary ligament rupture due to a bronchial artery aneurysm-induced hematoma: a case report

**DOI:** 10.1186/s40792-024-01810-3

**Published:** 2024-01-10

**Authors:** Yasuaki Tomioka, Eiji Yamada, Tsuyoshi Hyodo, Masahiko Muro

**Affiliations:** 1 Division of Thoracic Surgery, Department of Surgery, Fukuyama City Hospital, Fukuyama, Hiroshima Japan; 2 Division of Radiology, Department of Diagnostic Radiology and Interventional Radiology, Fukuyama City Hospital, Fukuyama, Hiroshima Japan

**Keywords:** Bronchial artery aneurysm, Bronchial artery embolization, Chest and back pain, Mediastinal hematoma, Massive hemothorax, Rupture, Angiography, Video-assisted thoracoscopic surgery

## Abstract

**Background:**

Bronchial artery aneurysm (BAA) is a rare vascular anomaly with the potential for serious complications, such as rupture leading to hemothorax or hemoptysis. Although bronchial artery embolization (BAE) is recognized as an effective intervention for ruptured BAA, video-assisted thoracoscopic surgery (VATS) is a minimally invasive approach for the treatment of associated hemothorax.

**Case presentation:**

A 73-year-old woman presented with a mediastinal hematoma from a ruptured BAA, causing bilateral hemothorax. Emergency angiography revealed a saccular BAA that was successfully embolized using a microcatheter and coil. Subsequent computed tomography revealed an expanding hemothorax managed by VATS, with 1400 mL of blood drained. During VATS, thoracoscopy revealed pulmonary ligament rupture, which was attributed to increased intramediastinal pressure. The patient was discharged eight days postoperatively with no complications. This case highlights the use of BAE and VATS in the management of mediastinal BAA rupture and massive hemothorax.

**Conclusions:**

BAE proved to be an effective strategy for the management of ruptured mediastinal BAAs. VATS is a valuable standby procedure for hematoma removal, but the indication should be carefully determined because of the risk of BAA re-rupture.

## Background

Bronchial artery aneurysms (BAAs), first described in 1930 in a patient with syphilis [1], are rare vascular entities with an incidence of less than 1% based on diagnosis by selective bronchial angiography [2]. Moreover, BAAs are usually asymptomatic, but their rupture can cause serious complications such as hemothorax and hemoptysis, leading to hemorrhagic shock [3].

Management of a ruptured BAA is a formidable challenge, and bronchial artery embolization (BAE) has consistently demonstrated efficacy in these scenarios [4]. Additionally, video-assisted thoracoscopic surgery (VATS) has emerged as a minimally invasive and advantageous approach for hematoma evacuation in cases of massive hemothorax secondary to BAA rupture [5].

Herein, we present the case of a 73-year-old woman who developed a mediastinal hematoma from a ruptured BAA that resulted in bilateral hemothorax. In this case, hematoma evacuation and mediastinal plural incision using VATS in addition to BAE were effective. Notably, our case also revealed a rupture of the pulmonary ligament, which was confirmed by thoracoscopy, and may be a common site of perforation when mediastinal hematoma perforates into the thoracic cavity.

## Case presentation

**Fig. 1:**
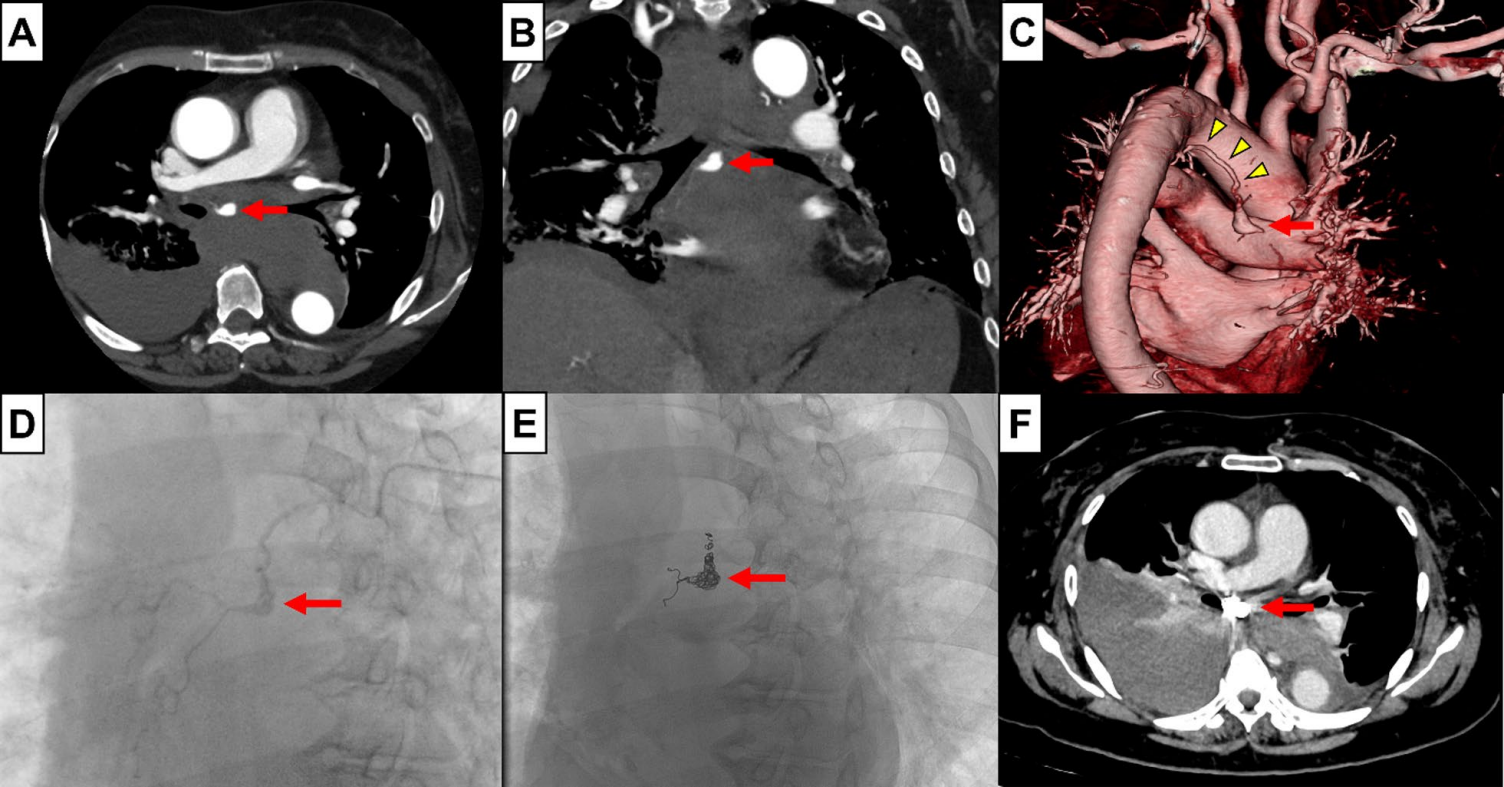
Imaging of the mediastinum. Computed tomography (CT) in the (**A**) axial and (**B**) sagittal planes shows a mediastinal BAA (red arrow) with an associated hematoma and right hemothorax. **C** Three-dimensional CT showing the long neck (yellow arrowheads) of the BAA (red arrow) and its origin from the descending aorta. **D** Bronchial arteriography showing a BAA (red arrow) in the right bronchial artery. **E** The BAA (red arrow) is successfully treated with catheter and coil embolization. **F** Subsequent CT image showing the treated BAA (red arrow) and expanded hemothorax. *BAA* bronchial artery aneurysm, *CT* computed tomography

**Fig. 2:**
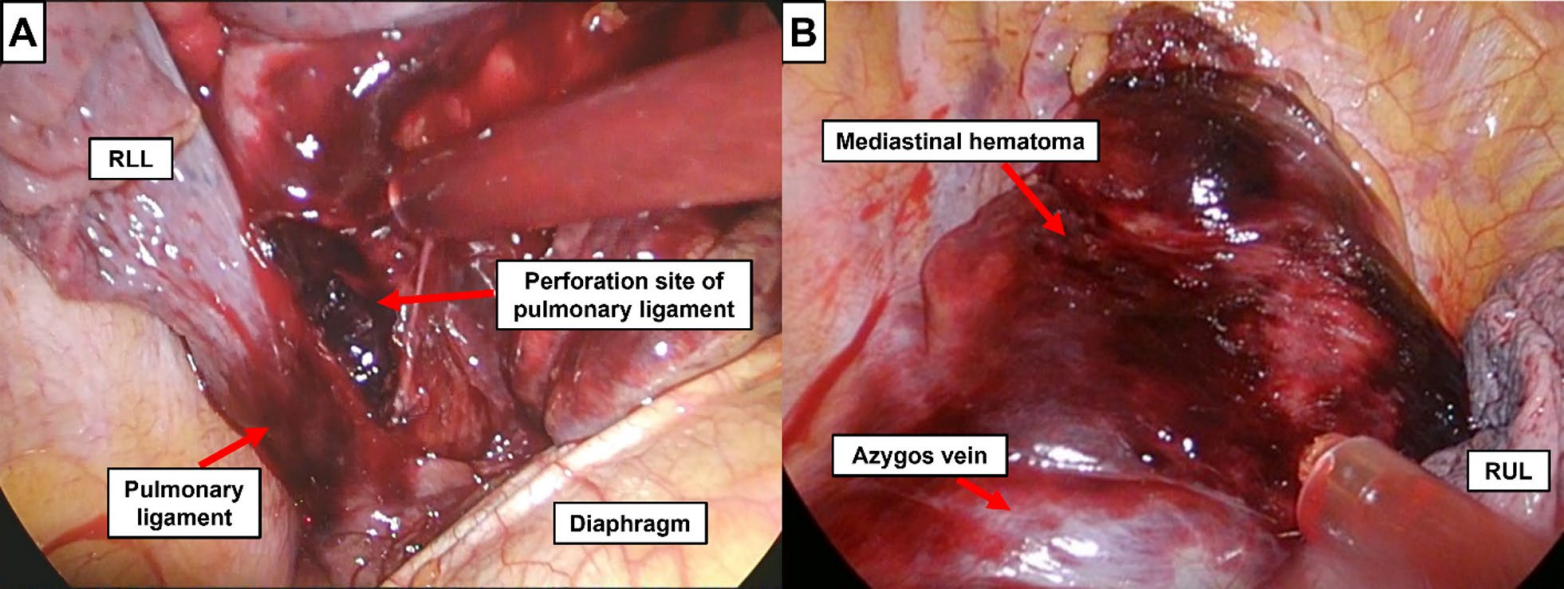
Intra-operative images. **A** A tear was observed on the ventral side of the pulmonary ligament near the hematoma. **B** Swelling from the intra-mediastinal hematoma was observed in the superior mediastinum. *RLL* right lower lobe, *RUL* right upper lobe

A 73-year-old woman presented to the emergency department of our hospital with sudden onset chest and back pain accompanied by dyspnea. Her medical history was only radiation therapy after partial mastectomy for left breast cancer. Contrast-enhanced computed tomography (CT) angiography revealed a mediastinal hematoma due to a 9.7 mm rupture of a mediastinal BAA originating in the aortic arch, with mild compression of the left atrium (Fig. 1A–C). Moreover, the rupture also resulted in acute bilateral hemothorax with greater right-sided involvement. Given the deteriorating pre-shock condition of the patient, emergency angiography was performed under local anesthesia. The right common femoral artery was punctured, followed by selective catheterization of the right bronchial artery using a 4Fr catheter. Selective bronchial arteriography revealed a saccular BAA originating from the proximal right bronchial artery (Fig. 1D). The BAA was embolized using a microcatheter, and its proximal and distal sides were successfully embolized using coils (Fig. 1E). After BAE, the patient's vital signs stabilized, but she required blood transfusions because of a drop in her hemoglobin level (from 13.1 to 8.9 g/dL). CT scan performed the following day revealed an expanding hemothorax in the right chest cavity and mild compression of the left atrium associated with a mediastinal hematoma. Additionally, the patient's respiratory status slowly deteriorated (from 0.5 to 4 L/min via nasal cannula), and VATS hematoma evacuation and mediastinotomy were scheduled two days after BAE (Fig. 1F). If the intrapleural hematoma is coagulated, thoracic drainage alone may not be sufficient to drain the intrapleural and mediastinal hematomas; therefore, we chose VATS, which ensures drainage. Surgery was performed with the patient in the left lateral decubitus position using one-lung ventilation. During the procedure, the hematoma was predominantly located on the ventral side of the pulmonary ligament and diaphragm. Moreover, a tear in the pulmonary ligament adjacent to the hematoma was identified as the site of the perforation (Fig. 2A). The mediastinal pleura on the cephalic side of the inferior pulmonary vein was stretched due to the swelling of the hematoma, which was also observed in the superior mediastinum. An incision was made in the mediastinal pleura over the azygos vein and below the tracheal bifurcation with great care not to touch the BAA (Fig. 2B) and approximately 1400 mL of blood was drained from the mediastinal hematoma. No surgical complications occurred, and the postoperative recovery was uneventful, with complete lung re-expansion. No further deterioration of vital signs was noted during the perioperative period, and the patient was discharged eight days after surgery.

## Discussion

This study highlighted several important clinical findings. First, the efficacy of BAE in managing mediastinal BAA ruptures was demonstrated. Second, VATS was instrumental in the management of massive hemothorax by facilitating hematoma evacuation. In addition, thoracoscopy confirmed that the increase in intramediastinal pressure due to the mediastinal hematoma precipitated rupture of the pulmonary ligament, culminating in a hemothorax.

BAA is a rare but potentially life-threatening condition that can lead to hemorrhagic shock if ruptured [3]. The exact etiology of BAA is unknown, but it has been attributed to increased blood flow in the bronchial arteries and focal weakening or damage to the vessel walls [6]. BAA can be congenital, associated with pulmonary sequestration or agenesis [7], or acquired, associated with atherosclerosis, inflammatory lung diseases, such as bronchiectasis, systemic diseases, or trauma. However, no predisposing etiology has been identified in approximately a quarter of reported cases. In our case, the etiology of the BAA was considered idiopathic because the patient’s medical history included only extra-chest wall radiotherapy after breast cancer. According to their different associated clinical symptoms, BAA is typically classified anatomically, either mediastinal or intrapulmonary [2], and the present case fell into the mediastinal type. The main symptoms of mediastinal BAAs are related to the compression or rupture of adjacent structures [8]. Most ruptured BAAs present with chest and back pain and hemothorax due to rupture of the pleural cavity or mediastinum. Current treatment options include surgical resection, transarterial embolization, and aortic stent grafting, followed by percutaneous embolization of the feeding vessels [3].

Rupture of the BAA within the mediastinum was the initial challenge in this case. Furthermore, the rupture created a significant hematoma that exerted pressure on the left atrium, destabilizing the patient's vital signs and threatening her overall health. BAE is a well-established, minimally invasive endovascular procedure with a high success rate [4]. However, its universal applicability remains controversial; aortic stent grafting may be more appropriate for aneurysms close to the bronchial artery opening or those with a short neck [9]. In our case, BAE was indicated by the considerable distance between the proximal bronchial artery and aneurysm. Recently, thoracic stent grafting in combination with percutaneous embolization has been used to treat mediastinal BAAs in which embolization alone is anatomically inappropriate [10].

Management of the resultant hemothorax was the second most crucial aspect of our intervention. Although some reports have recommended immediate thoracoscopic hematoma evacuation after BAE [5], we did not plan VATS immediately after BAE because of successful control of bleeding after BAE. However, a CT scan performed the day after BAE showed mild compression of the left atrium due to a posterior mediastinal hematoma, and the patient's respiratory status worsened with hemothorax expansion. We considered that not only evacuation of the intrapleural hematoma, but also incision of the mediastinal pleura was necessary. If the intrapleural hematoma was coagulated, we concluded that thoracic drainage alone might be insufficient to drain the intrapleural and mediastinal hematoma, and that VATS was appropriate. VATS plays a pivotal role in draining the hemothorax, ensuring lung re-expansion, and minimizing potential complications. VATS allows excellent visualization of the pleural space and is beneficial in draining pleural effusions and improving respiratory function compared to chest tube drainage [11]; If there is hemodynamic or respiratory deterioration due to hematoma expansion even after BAE with or without chest drainage, hematoma evacuation by VATS and mediastinotomy may be considered.

A unique observation in our case was rupture of the pulmonary ligament, which was confirmed by thoracoscopy. The increased intramediastinal pressure due to the mediastinal hematoma probably caused this rupture, leading to the perforation of the hematoma in the thoracic cavity. This observation may suggest that the pulmonary ligament is the common site of perforation when mediastinal hematoma perforates into the thoracic cavity.

## Conclusions

BAE remains an effective strategy for the management of ruptured BAAs. In addition, VATS is an effective procedure for hematoma evacuation, but the indication should be carefully determined because of the risk of BAA re-rupture.

## Data Availability

Not applicable.

## Abbreviations

BAA: Bronchial artery aneurysm
BAE: Bronchial artery embolization
CT: Computed tomography
VATS: Video-assisted thoracoscopic surgery

## References

[1] Wilson SR, Winger DI, Katz DS (2006). CT visualization of mediastinal bronchial artery aneurysm. Am J Roentgenol.

[2] Tanaka K, Ihaya A, Horiuci T, Morioka K, Kimura T, Uesaka T (2003). Giant mediastinal bronchial artery aneurysm mimicking benign esophageal tumor: a case report and review of 26 cases from literature. J Vasc Surg.

[3] Misselt AJ, Krowka MJ, Misra S (2010). Successful coil embolization of mediastinal bronchial artery aneurysm. J Vasc Interv Radiol.

[4] San Norberto EM, Urbano Garcia J, Montes JM, Vaquero C (2018). Endovascular treatment of bronchial aneurysms. J Thorac Cardiovasc Surg.

[5] Kim HJ, Son HS, Cho SB, Kim KT (2013). Development of a life-threatening mediastinal hematoma from a ruptured bronchial artery aneurysm. J Thorac Cardiovasc Surg.

[6] Hoffmann V, Ysebaert D, De Schepper A, Colpaert C, Jorens P (1996). Acute superior vena cava obstruction after rupture of a bronchial artery aneurysm. Chest.

[7] Mizuguchi S, Inoue K, Kida A, Isota M, Hige K, Aoyama T, Ishikawa T (2009). Ruptured bronchial artery aneurysm associated with bronchiectasis: a case report. Ann Thorac Cardiovasc Surg.

[8] Hall RJ, Miller GA, Kerr IH (1977). Ruptured bronchial artery aneurysm mimicking aortic dissection. Br Heart J.

[9] Lin JL, Ji YY, Zhang MZ, Tang Y, Wang RL, Ruan DD (2022). Rare cases of bronchial aneurysm and comparison of interventional embolization in the treatment of true bronchial aneurysm and pseudobronchial aneurysm. Front Cardiovasc Med.

[10] Song M, Wu H, Jiang J, Wang G, Wu S, Ge T (2016). Mediastinal bronchial artery aneurysm treated with aortic stent and embolization: case report. Int J Clin Exp Med.

[11] Manlulu AV, Lee TW, Thung KH, Wong R, Yim AP (2004). Current indications and results of VATS in the evaluation and management of hemodynamically stable thoracic injuries. Eur J Cardiothorac Surg.

